# Ensuring Reliable Network Communication and Data Processing in Internet of Things Systems with Prediction-Based Resource Allocation

**DOI:** 10.3390/s25010247

**Published:** 2025-01-04

**Authors:** Weronika Symbor, Łukasz Falas

**Affiliations:** Department of Computer Science and Systems Engineering, Faculty of Information and Communication Technology, Wrocław University of Science and Technology, 50-370 Wrocław, Poland

**Keywords:** reliability, resource allocation, quality of service, IoT, machine learning, network traffic prediction

## Abstract

The distributed nature of IoT systems and new trends focusing on fog computing enforce the need for reliable communication that ensures the required quality of service for various scenarios. Due to the direct interaction with the real world, failure to deliver the required QoS level can introduce system failures and lead to further negative consequences for users. This paper introduces a prediction-based resource allocation method for Multi-Access Edge Computing-capable networks, aimed at assurance of the required QoS and optimization of resource utilization for various types of IoT use cases featuring adaptability to changes in users’ requests. The method considers the current resource load and predicted changes in resource utilization based on historical request data, which are then utilized to adjust the resource allocation optimization criteria for upcoming requests. The proposed method was developed for scenarios utilizing edge computing, e.g., autonomous vehicle data exchange, which can be susceptible to periodic resource demand fluctuations related to typical rush hours, predictable with the proposed approach. The results indicate that the proposed approach can increase the reliability of processes conducted in IoT systems.

## 1. Introduction

**Figure 1:**
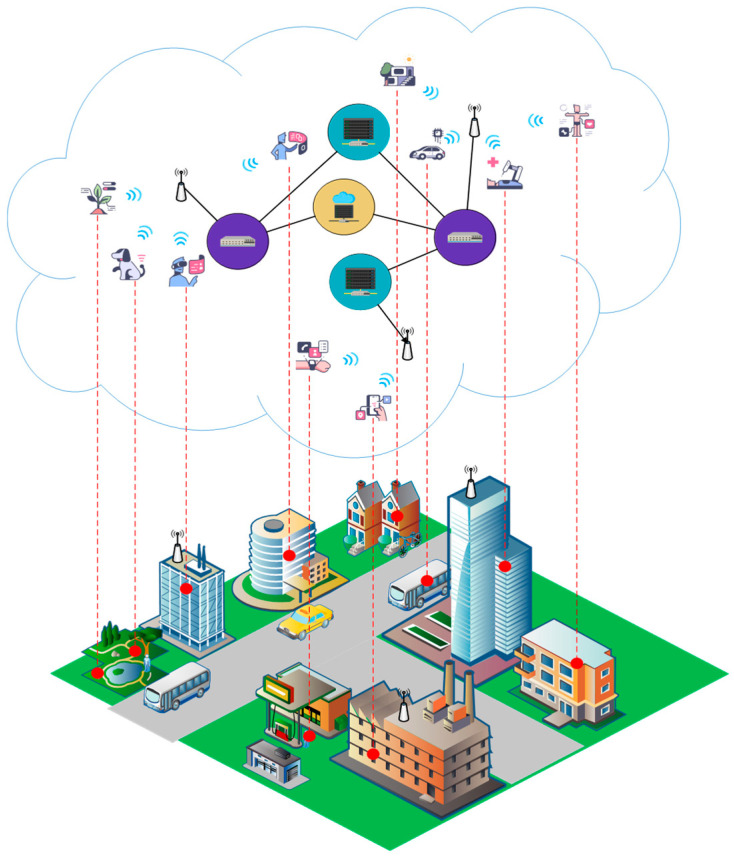
Next-generation network use cases.

In recent years, an increasing development of distributed Internet of Things (IoT) and other next-generation network (NGN) applications has been observed. Beyond the typical IoT telemetry scenarios, these new applications incorporate emerging use cases (Figure 1) focused on the real-time transfer of high-fidelity data (e.g., real-time footage from drone cameras over IP networks), remote control of industrial machines and unmanned air, land, and naval vehicles, autonomous driving performed by artificial intelligence systems, developing and maintaining virtual environments acting as digital twins in industrial applications based on real-world sensor data, high-quality multimedia data streaming in near-real-time, and various e-health scenarios including telesurgery and remote patient monitoring [1]. These new usage scenarios lead to new challenges emerging from their increasing adoption. As systems interact with real-life objects and people, reliability concerns emerge, for which we need broader insights. Failure in the delivery of the required network and computing performance, resulting, for instance, in greatly increased latency or an inability to properly handle user requests, can also introduce significant issues to users and the systems’ ability to perform according to the defined requirements.

An example of a system prone to such challenges is an unmanned air vehicle (UAV) remote control system over mobile networks like 5G, which is studied in some research focused on building drone prototypes [2] and proposing methods for networks which support UAV use cases [3,4]. Mobile network performance may suffer from various types of events like an increased number of clients in the given area, lack of network resources in the new network cell entered by the UAV, or a lack of network resources due to spikes in network usage by other users. If such an event occurs, the reliability and safety of such a UAV will suffer, because control over the UAV may be lost and, as a result, it can crash. There are various possible outcomes of such an event, ranging from damage to the UAV itself, through to damage to the environment at the crash site, and to possible injuries to people who were at the crash site when the UAV fell. Furthermore, even if there is no damage, such a UAV and the data stored on it can be stolen after the crash or simply lost, e.g., during a mission in a remote location. Considering these facts, it seems reasonable to view performance and reliability issues as one of key challenges in modern distributed systems, especially in IoT systems.

The aforementioned new challenges present in modern IoT systems require a new approach to the distributed systems architecture, emphasizing the need for quality of service (QoS) assurance mechanisms that will guarantee compliance with the defined functional and non-functional requirements of the systems [5]. Due to the distributed nature of such systems, these solutions must be based on functions and solutions present in the networking and computational infrastructure provided for such systems. However, with the introduction of new concepts, like fog computing and extending cloud services, to the network edge in order to enable service providers to deploy their services closer to the end users and thereby decrease latency, new possibilities for QoS assurance are emerging. This concept thrives especially in mobile networks in the form of Multi-Access Edge Computing (MEC), which is specifically designed for mobile networks and closely associated with 5G networks, focusing on the migration of computing and network traffic from centralized cloud services to the edge of the mobile network, closer to the end user [5]. The MEC concept aims to provide close proximity, ultra-low latency, a high bandwidth, and virtualized services to users wherever they are utilizing them.

The MEC architecture provides mechanisms for the migration of computing and network traffic in the infrastructure. However, the architecture itself does not provide methods for managing and orchestrating the location of services and utilization of network links in a manner that assures the required QoS levels for various services [6]. This process can be conducted by service providers, which manually deploy specific or currently popular services to the region in given locations. It can also be conducted semi-automatically by providing predefined pools of computing and networking resources that can be utilized to deploy services on-demand on the basis of their current usage [7]. As the current research indicates, the key challenges in delivering proper QoS assurance are related to latency sensitivity, resource awareness, job affinity, load awareness and isolation, application characteristics, and application-specific QoS requirements [8,9]. However, in order to provide capabilities for adaptable QoS assurance overcoming these challenges, new resource allocation methods are needed. 

The main motivation for this research was the consideration that with the development of new IoT use cases, some of them may require hard QoS guarantees to ensure their correct execution. Such use cases may be related to the automotive domain. Even though, currently, most autonomous vehicles rely on their own sensors, the idea guiding their further development is that the traffic flow in cities can be optimized by enabling interconnectivity and information exchange between vehicles, as well as by introducing mechanisms that can orchestrate the traffic by providing cars with instructions, as was shown in research discussing cooperative, connected, and automated mobility (CCAM) [10] and vehicular cloud computing (VCC) [11] challenges as well as research on specialized data exchange and digital-twin-based intent networks for autonomous traffic [12]. However, the networking and computational infrastructure for such a use case must have a stable and dependable performance, to assure the desired system operability. The infrastructure for such use cases may be a typical shared network that utilizes a best-effort policy, which can be susceptible to traffic overloads, resulting in QoS deterioration when other use cases emerge, such as high-quality video streaming by numerous users in the area. Then, such networking mechanisms will probably not suffice for mission-critical use cases. On the other hand, an isolated network can be deployed for use cases with resources dedicated to a single type of use case. However, such a solution will probably not be suitable for scenarios that have a variable resource demand, which can fluctuate due to the time of the day or similar events, because such infrastructure can be fully utilized at one point in time but it can also be underutilized for long periods. 

This research was aimed at providing a middle-ground solution that offers both a reasonable level of QoS assurance as well as possibilities for effective utilization of resources normally used, for critical applications when they are underutilized. In an automotive use case related to car traffic in the city, it can be assumed that increased traffic periods, when more networking or computational resources are required, may be predicted through the utilization of statistical or machine learning algorithms, so resource allocation may be conducted in a way that during rush hours, a large part of the infrastructure is dedicated to those critical use cases. At the same time, when the traffic is lower and requires fewer resources, a larger part of the infrastructure can be utilized for lower-priority traffic generated by other users, e.g., high-quality streaming in video on-demand services. This adaptability of the proposed method ensures that the system can dynamically respond to changes in end users’ requests, optimizing the utilization of available resources.

With that as the concept, this paper proposes a prediction-based resource allocation method aimed at enabling the assurance of the required QoS levels for various types of IoT use cases and autonomous adaptability to changes in end users’ requests based on request analysis and machine learning methods, effectively optimizing the utilization of available resources. In the proposed approach, a network segment between the cloud gateway and end users is modeled as a graph, consisting of nodes with specified resources capable of both computing and routing network traffic or just routing traffic. Nodes considered as network leaves represent end users or their groups, and edges represent the network links with specified available networking resources. This representation is utilized to optimize both the deployment of services on available computing nodes and the allocation of network resources on the links between the nodes in such a way that ensures proper QoS levels for all traffic that can be handled by the given network resource capacity. The optimization is conducted on the basis of the current network utilization and predicted changes in the utilization and based on historical training data by machine learning algorithms. The main benefits and innovations of the proposed approach are adaptability and QoS assurance based on traffic prediction, increased efficiency of network and computational resource utilization, increased reliability of user request processing, and decreased networking and computational infrastructural costs.

The proposed method was developed especially for scenarios utilizing mobile networks offering MEC capabilities, e.g., 5G networks. However, after some adaptations, the proposed solution can probably also be utilized in other networks that incorporate network edge processing mechanisms similar to the aforementioned MEC network architecture, those which offer service migration functions and edge processing capabilities on servers deployed closed to the end users. The proposed approach will be discussed in detail in the following sections. The next section presents a brief description of related works and the current state of the art in the field of resource allocation for networks utilizing the MEC architecture. Section 3 discusses the next-generation network model, followed by a description of the proposed prediction-based resource allocation process and a formal definition of the resource allocation optimization problem. Section 4 introduces the general resource allocation algorithm and two network traffic prediction algorithms utilizing Autoregressive Integrated Moving Average (ARIMA) and Long Short-Term Memory (LSTM) neural network approaches. Section 5 discusses the experimental verification of the effectiveness of the resource allocation method, incorporating a comparison between two network traffic prediction approaches. Finally, Section 6 presents the conclusion regarding the effectiveness and applicability of the proposed approach and discusses potential future research in this field.

## 2. Related Works

**Figure 2:**
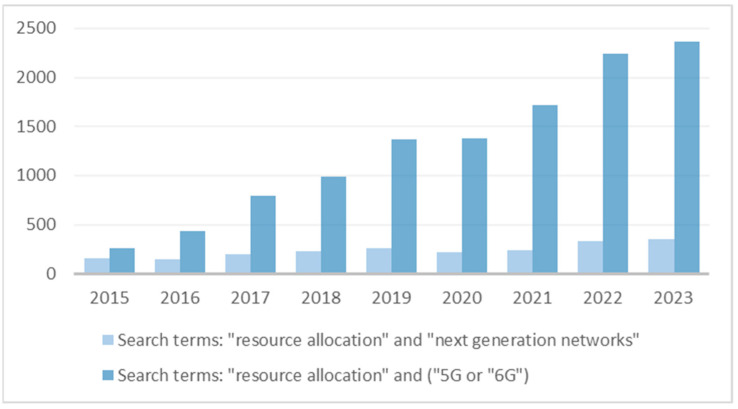
Number of resource-allocation-related publications in the years 2015–2022, on the basis of searches in the MDPI, IEEE Xplore, and ACM Digital Library databases.

Prediction-based resource allocation presents a joint problem, combining the challenges of both resource allocation in network systems as well as network traffic prediction. Given that resource allocation is one of the fundamental issues in network systems, many different solutions to this problem are discussed in research papers. Some involve static resource allocation, where resources are allocated based on a predetermined set of rules and do not change during system operation [13]. Other methods include a dynamic resource allocation strategy, which involves adjusting allocations in real-time in response to changing conditions or requirements, as well as hybrid approaches that combine both of these strategies. Resource allocation can be classified into reactive and proactive approaches [14]. In reactive resource allocation, decisions are made immediately after the user initiates a request [15]. In turn, the proactive approach takes into account future characteristics, such as network traffic patterns and demand for specific network resources. A proactive approach can, therefore, help make effective decisions about resource allocation before an event occurs, thus adapting the network to future expected needs. The resource allocation issues in the context of next-generation networks are often discussed in scientific publications. Analysis of the number of publications in the MDPI, IEEE Xplore, and ACM Digital Library databases (Figure 2) indicates a growing trend and the current importance of resource allocation in the context of the next-generation network.

Recent publications and reviews show that resource allocation in the 5G network is a key element ensuring effective access to services and applications for end users, discuss various methods and techniques for resource allocation, and indicate the need for future research in this field [16]. There is also an indication that the most important metrics for this problem are related to network throughput, energy efficiency, and computing power. Extant research in this field utilizes linear programming methods [17], algorithms based on swarm intelligence [18,19,20], game theory approaches [21,22], and genetic modeling [23,24,25]. The specific nature of next-generation networks, as well as varied requirements for the quality of services, may cause the problem to be classified as NP-hard [26]. Accordingly, linear programming methods may be suitable for small network infrastructures. Meanwhile, in the case of more complex problems, an approach based on heuristics or the search for Pareto optimal solutions is usually used.

In this paper, the second important topic addressed is the problem of network traffic prediction. In recent research, this is often solved by using supervised machine learning methods, mainly with classification and regression methods. Classification algorithms can be utilized to label requests according to their identified traffic class. Next, regression-based methods are utilized to forecast the demand for particular resources in a designated future time window. Recently, many solutions to the network traffic prediction problem have focused on the utilization of artificial neural networks, including the Q-learning algorithm and deep reinforcement learning [27]. One example of such research uses recurrent neural networks consisting of Long Short-Term Memory (LSTM) [28] and then predicts traffic patterns one week ahead. Another piece of work [29] addresses the problem of dynamic bandwidth allocation of a 5G transport network segment, using an approach that consists of two stages: traffic forecasting using an LSTM-based method, and then bandwidth configuration. The experimental results indicate that the use of LSTM allows for achieving a high accuracy in predicting network traffic. This enables the dynamic allocation of resources to services with different priorities, while taking into account the requirements for different levels of quality depending on the three classes of services (eMBB, URLLC, and mMTC), resulting in an improved service quality and user experience across the system. In article [30], three different machine learning and deep learning models are used to predict the network load in the architecture of 5G mobile network services—a One-Dimensional Convolutional Neural Network (1D-CNN), a Multilayer Perceptron Regressor (MLP), and a K-Nearest Neighbors (KNN) regressor—combined with the Ensemble Learning (EL) method using the bagging technique. The generated dataset included five different device types and three different categories of bandwidth requirements for each device. Ensemble learning allowed the predictions to be combined by averaging, thus creating the final prediction result. The measures used allowed for the evaluation of the results, which showed that the solution based on ensemble learning had the best performance. According to the authors, although the regression models produced relatively good results, their performance may be worse when implemented in real network conditions. In [31], three different classes of predictors were compared in solving a real-time traffic prediction problem: classical time series, artificial neural networks, and wavelet transform-based predictors. They were then evaluated using real network data. The results indicated that DES and ARMA predictors show high accuracy with minimal energy consumption, while predictors based on an artificial neural network, despite having good accuracy, required a high computing power.

Considering the state of the art in the field of resource allocation in next-generation networks, as well as developments in network traffic prediction, this paper proposes a prediction-based resource allocation approach, utilizing both heuristic methods for resource allocation and machine-learning-based network traffic prediction. The combination of these solutions should enable the practical utilization of the proposed approach in modern next-generation networks, especially in 5G networks.

## 3. Prediction-Based Resource Allocation in IoT Systems

The main concept behind the proposed resource allocation method is that on the basis of historical network traffic analysis, the future network load can be anticipated for various types of traffic. The changes in different network traffic types can be linked to various factors like the time of day, number of connected users, previous changes in network traffic, and others. As performing a prediction on the individual request level is a difficult task, in the proposed approach, a share in the total network traffic for each of the identified traffic types is predicted. This approach introduces much less of a computational overhead and it is more manageable and easier to practically implement in the NGN infrastructure.

The prediction of the network traffic share for each type of identified traffic is utilized in the process of prediction-based resource allocation, which enables increased security and reliability through QoS assurance, while optimizing the overall NGN load. The key idea for this approach is that the information about the anticipated shares of each traffic type, which can differ in priority and typical computational and network resource usage, can help the resource management system in the NGN to prepare for upcoming requests and preemptively adjust parameters in the optimization criteria in such a way that prepares the infrastructure for anticipated changes in resource demand from incoming user requests. Such adjustments in the optimization criteria should result in an overall better quality of service for all accepted user requests and provide more reliable computational and networking services, especially for mission-critical requests processed in NGNs. Also, this approach can help to ensure the reliability and security of high-priority traffic. On the basis of future traffic prediction, the prediction-based resource allocation method will first try to allocate the resources for high-priority traffic, according to the prediction regarding the amount of that traffic in future requests. Then, the resources are allocated for traffic types with a lower priority, as long as there are free resources. Yet, the proposed approach currently does not have mechanisms for preempting requests; hence, it is possible that due to prediction inaccuracy, high-priority traffic will not be served correctly.

### 3.1. Next-Generation Network Model

The proposed prediction-based resource allocation approach is aimed at providing new capabilities for NGN systems and applications. In its discussion, this paper focuses mainly on mobile network scenarios utilizing 5G technologies; however, this approach can be utilized in any similar NGN infrastructure. The proposed approach focuses on modeling the network segment closest to the end user due to thrive, to deliver enablers that realize the promise of Enhanced Mobile Broadband (eMBB), Massive Machine-Type Communication (mMTC), and Ultra-Reliable Low-Latency Communication (URLLC), which is only possible if services are deployed close to the user, according to the concepts of Multi-Access Edge Computing [32].

The proposed solution models the network segment as a graph with four distinct types of nodes interconnected with edges. The *root* represents the main node of the segment acting as a gateway to other networks. In many cases, it will act as a connection point to cloud services; hence, in this paper, it is also referred to as a cloud gateway. The important feature of this node is that it is an abstract node, and it is not related to any physical device in the network. Also, there can be only one *root* node in the graph; hence, if there are multiple physical network devices that can act as gateways, they should all be connected to the *root* node, representing the connection point to the external networks.

Devices present in the network that can provide edge computing capabilities are considered *active* nodes. These are nodes that can be utilized for services that should be deployed closer to the end user. These nodes are crucial for the resource allocation optimization process as each of them is described by the available computing resource pool (e.g., available CPUs, GPUs, RAM), which is taken into consideration during the optimization process. The process of resource allocation for these nodes is performed by computing resource reservation for each user request in a way that assures the required quality of service.

Typical network devices that lack computing capabilities are considered *passive* nodes in the graph network model. These nodes are only responsible for network traffic routing. In the proposed resource allocation approach, it is assumed that they can handle all network traffic as long as the directly connected network links’ capacity is not exceeded.

The *leaf* nodes are the final type of node in the proposed network model. Similar to the *root* node, they can be considered abstract nodes that do not have to be mapped to a single device. The *leaf* node represents a user request source that requires the reservation of computing or networking resources, or both. A *leaf* node can represent a single device or a set of devices, sending requests related to different types of traffic. In the proposed model, each *leaf* node is linked to a prediction model, which indicates what type of requests we can anticipate in the near future.

Network links in the infrastructure are represented as *edges* in the graph network model. Each of the *edges* is described by a set of parameters representing its capacity and current load. Previous research work focused mainly on the available bandwidth, delay, and security level. However, the set of parameters describing network links can easily be adjusted in future research works.

**Figure 3:**
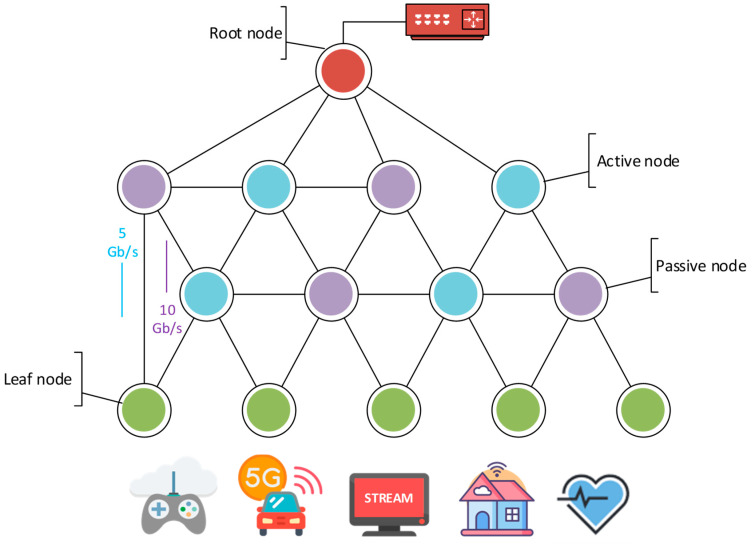
Next-generation network model example.

Figure 3 presents a sample of such a graph network model. The *root* node is depicted in red, the *leaf* nodes are green, the *active* nodes are blue, and the *passive* nodes are violet. This sample represents a simple distributed MEC infrastructure, offering both networking and computing capabilities to end users, utilizing different types of services like cloud gaming, video streaming, e-health, IoT traffic, and machine-to-machine communication between autonomous vehicles. In such an infrastructure, key services that require a low delay (e.g., autonomous vehicles) can be placed closer to the end user. On the other hand, high-bandwidth services like video streaming can be deployed in a way that enables network offloading on edges closer to the root, which, due to their localization in the infrastructure, aggregate the network traffic from nodes closer to the end user. As a result, this can free up bandwidth for user requests exclusively utilizing cloud services available via the root node and can lead to an overall increase in the quality of service perceived by end users.

While this topic is out of the scope of the current paper, it is worth noting that the proposed solution can be applied beyond a single segment of the network. For larger networks, each individual segment can be managed by a local optimization method and all of the excess traffic can be forwarded through the cloud gateway. This may occur in situations in which a local network segment runs out of computing resources to handle requests, which may be caused by incorrect network traffic prediction leading to bad resource allocation decisions or simply by exhausting the available infrastructural capacity. Then, the system may utilize the described policy to forward the requests that cannot be handled via the gateway instead of rejecting them. Such an approach may be taken at the expense of the QoS during its execution; however, it is a scenario that can be considered if the resource allocation mechanism extends beyond a single network segment and if the priority is to handle all the requests, instead of rejecting requests that cannot be handled in line with QoS requirements. 

**Figure 4:**
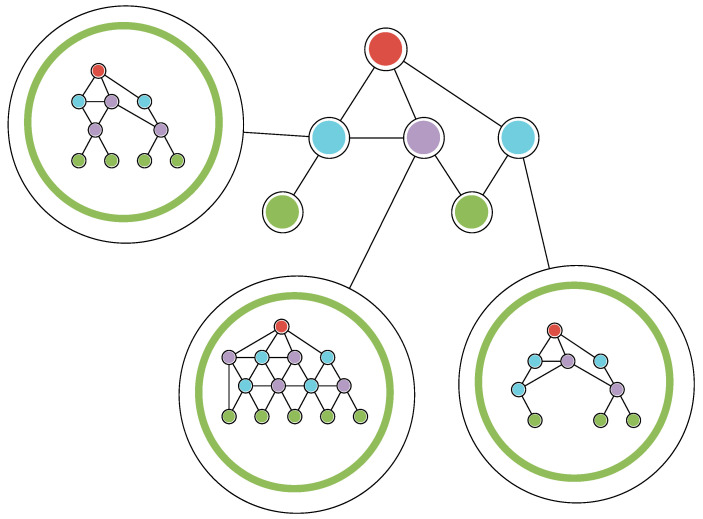
Next-generation network model with encapsulated network subsegments.

All excess requests relayed by the cloud gateway can be viewed higher up in the hierarchy simply as user requests in the network. The whole segment that forwards the requests can then be encapsulated as a subsegment and considered a leaf in a network model higher up in the network hierarchy, thus traffic from this subsegment can be optimized in the same way as from other leaf nodes (Figure 4).

### 3.2. Prediction-Based Resource Allocation Process

The proposed prediction-based resource allocation approach assumes that all requests in the network can be tracked and classified into one of the predefined traffic types. This approach also utilizes the reservation of resources for user requests, similar to the Integrated Services (IntServ) architecture. As a result, user requests are processed as long as there are available computational and networking resources in the system to guarantee end-to-end connectivity and processing in the system. All requests that would exceed the available resources at any point in the infrastructure are rejected by the system. It is worth noting that in this approach, such a system can have an additional pool of resources serving other types of traffic, which can be utilized to handle rejected resources without any QoS guarantees. However, that scenario is not in the scope of this paper.

One of crucial challenges in systems enforcing such a strict QoS policy is to provide resource allocation mechanisms that maximize the utilization of infrastructural resources while minimizing the number of rejected requests. The difficulty of this task is directly related to the variety of required networking and computational resources in different user scenarios. Without proper planning, such a network system can easily become unusable as a result of overloading certain parts of the infrastructure. In contrast, other parts of the infrastructure can still offer free computing and networking resources but are virtually unavailable due to excessive utilization of some network links or improper deployment of services, leading to bottlenecks. Such bottlenecks significantly deteriorate the quality of resource allocation and the capability of ensuring the required QoS for user requests. In the case of resource allocation, such bottlenecks can effectively isolate a large part of the computing and networking resources. Then, the resource allocation algorithm will not be able to allocate resources on certain computing nodes and network links, which are unreachable due to the bottleneck on one of the links on the path leading to those resources. As a result, the available computational and networking capacity of a system can be effectively decreased, even though there are unutilized resources that could have been allocated. The described situation can also result in decreased QoS assurance capability. Sometimes, the bottleneck can lead to a situation in which some network links (e.g., links offering a high level of security) or computing nodes offering specialized computing infrastructure (e.g., GPUs) are unreachable, despite the fact that they could still offer free processing power. In order to overcome this challenge, this paper proposes the utilization of prediction methods, in order to provide resource allocation algorithms with ahead-of-time information regarding the dynamic of changes in user-generated network traffic.

**Figure 5:**
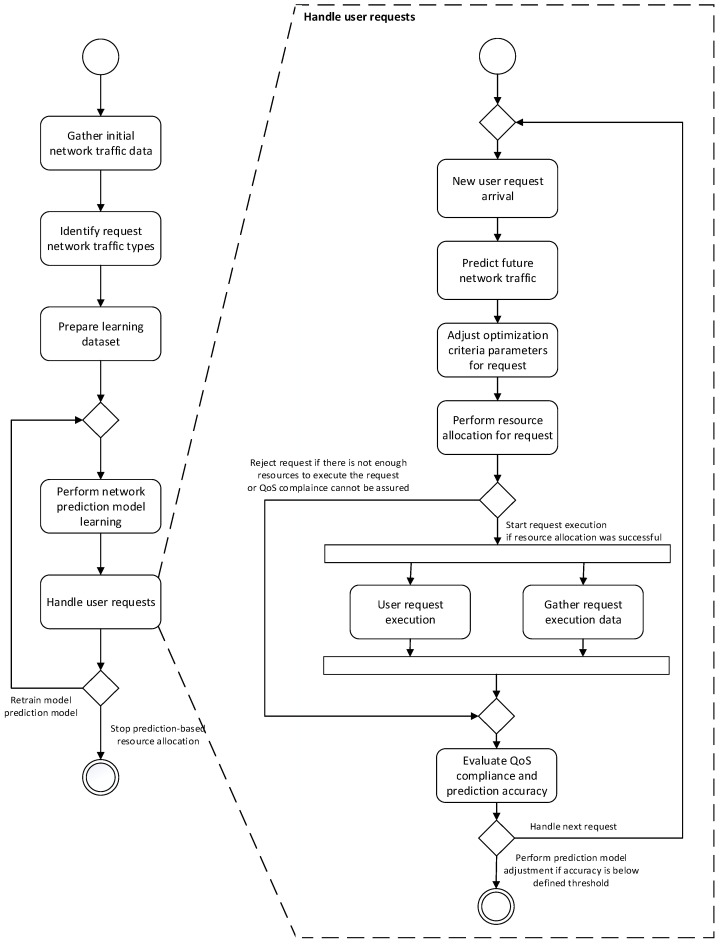
Prediction-based resource allocation process.

The resource allocation process in the proposed approach starts with request data gathering and analysis (Figure 5). On the basis of the initially gathered data describing user requests, the prediction model is built during the learning process, which is aimed at indicating shares of different types of network traffic in future requests. Following the completion of this process, this prediction model is then utilized in the resource allocation process. During the reservation of resources for each new user request, the resource allocation method utilizes the model to predict the shares of different types of network traffic in future requests. Considering the requests’ traffic types, priority levels, and required resources, and predictions regarding the future user requests’ network traffic types and their QoS and resource requirements, the resource allocation algorithm adjusts the values of optimization parameters (e.g., adds additional cost to the fitness function in order to enforce the desired resource allocation or performs a pre-reservation of computing and networking resources on certain edges) in such a way that it anticipates future user requests. After the reservation is made, the availability of resources in the graph network model is updated and request execution is performed. Following the reservation and request execution, the data related to handled user requests are stored for future tuning of the network traffic prediction algorithm, which can be conducted when the prediction accuracy falls below the defined threshold.

Excess network traffic or prediction model failures resulting in inaccurate prediction can lead to a situation in which resources available in the MEC infrastructure will be exhausted. If such an event occurs, the request is rejected and stays unhandled. While not ideal, this policy was utilized in our research evaluation, and the number of rejected requests resulting from different types of prediction models can be observed in our research data.

One of the possible alternative policies, which was not experimentally verified in this research, incorporates the utilization of the cloud gateway defined in this infrastructure. If the prediction is incorrect and the available computing resources in the network segment managed by the resource allocation algorithm are insufficient to handle the requests, but there are still available networking resources, the requests can be routed via the cloud gateway to the cloud computing infrastructure, where they can be processed. This can introduce the risk of not delivering the required QoS. However, it is a viable option for requests in which execution is crucial, even with the lowered QoS.

In order to verify the proposed prediction-based resource allocation process, an accurate network traffic prediction method and the resource allocation method itself must be developed. This paper focuses on the introduction of the general concept for this approach, while the experimental research is still preliminary and focuses on the verification of this concept and its potential applicability in real-life scenarios. Accordingly, several assumptions were made in order to bound the resource allocation optimization problem. The assumptions made were the following:

1. Each link has a certain quantity of communication resources that enable data transmission in the network, while only active nodes have computational resources available for network service calls. Additionally, a limited availability of network resources is assumed.
2. Successful resource reservation for a given request requires the availability of resources both in the global resource pool (considered as all available networking and computing resources in the analyzed network segment) and in the constrained resource pool provided for a given type of network traffic.
3. If it is impossible to guarantee network resources, the traffic is rejected (e.g., if there is an insufficient bandwidth to handle the traffic, or an insufficient quantity of computing resources to fulfill a given request).
4. The considered network accepts exactly one request per unit of time.
5. Each traffic class has information about the computing node needed to fulfill a given request.
6. It is assumed that the data regarding the required QoS parameters are known a priori and do not require any additional action to read them from external data storage.
7. Requests are handled by routing and allocating appropriate resources from the source (leaf node) to the root node and then back to the leaf node that made the request.
8. It is assumed that as part of traffic management, it is possible to divide the designated route into sub-routes, dividing the resources required to handle traffic in fixed proportions.

The request handling process in this scenario will require handling a queue of traffic requests over time. This process includes routing for individual requests, taking into account QoS requirements for different traffic classes and the presented assumptions. The overall goal of the process is to ensure the maximization of resource utilization, while minimizing the number of rejected requests and ensuring the required QoS for accepted requests.

### 3.3. Optimization Problem Formulation

The formal mathematical model for the resource allocation problem is built upon the proposed next-generation network model and the aforementioned assumptions regarding the prediction-based resource allocation process. The main problem in this task is to effectively allocate resources to requests coming to the system while keeping within the constraints resulting from the availability of networking and computing resources and requests’ QoS requirements. It should be emphasized that the presented model is still a preliminary approach to the prediction-based resource allocation problem, intended to outline the key concepts analyzed within the scope of this paper. For the concepts discussed in this paper, the following definitions and notations are used:

- A graph network model is defined as follows:

$$G=(N,L,W_{K},W_{O})$$

where

N: set of network nodes, $N=\left\{N_{1},N_{2},\ldots ,N_{V}\right\}$, where V is the total number of nodes;
L: set of network links, $L=\left\{L_{1},L_{2},\ldots ,L_{E}\right\}$, where E is the total number of links;
$W_{K}$: weights representing the maximum communication resources for the links;
$W_{O}$: weights representing the maximum computational resources for the nodes.

- Requests, traffic classes, and QoS parameters are defined as follows:

C: set of traffic classes, $C=\{c_{1},c_{2},\ldots {,c}_{R}\}$, where R is the total number of classes;
Z: set of requests, $Z=\left\{z_{1},z_{2},\ldots ,z_{M}\right\}$, where M is the total number of requests;
$c\left(z\right):$ traffic class assigned to request z, z∈Z;
$Q(c_{r})$: the QoS parameter vector, for the request identified as $c_{r}$ traffic class (may represent, for instance, the minimum requirements for bandwidth or RAM), where

$Q_{K}\left(z\right)$: vector of QoS parameters related to communication resources;

$Q_{O}(z)$: vector of QoS parameters related to computing resources;

$Q_{s}(z)$: vector of QoS parameters related to the required level of security.

The formulation of decision variables, objective functions, and constraints that make up the mathematical approach to the problem of resource allocation is presented below. Decision variables determine what decisions can be made within the resource allocation problem. The objective function indicates the criteria to be maximized or minimized, while the constraints ensure that the solution complies with specific requirements, such as not exceeding the available resource pool.

In the proposed approach, the following decision variables were identified:

- $x_{n,z}$: computing resources reserved for request *z* in node *n*;
- $y_{l,z}$: networking resources reserved for request *z* in link *l*;
- $s_{l}$: security level offered by link *l.*

The optimization problem is formulated with hard constraints, applied to both nodes and network links within the defined NGN model, as well as the QoS requirements, ensuring their strict and uncompromising fulfillment:

- For each active node, the total resources allocated cannot exceed the maximum resources:
  $$\sum_{z\in Z}x_{n,z}\leq W_{O}\left(n\right),\;\;\forall n\in N$$
  where $W_{O}\left(n\right)$ defines the maximum computing resources available for node n.
- For network links with defined networking resources:
  $$\sum_{z\in Z}y_{l,z}\leq W_{K}\left(l\right),\;\;\forall l\in L$$
  where $W_{K}\left(l\right)$ defines the maximum networking resources available for the network link l.
- For each request, the allocated computing resources on the node handling the request must match the resources required to ensure QoS requirement compliance:
  $$\sum_{n\in N}x_{n,z}=Q_{O}\left(z\right),\;\;\forall z\in Z$$
- For each network link *l* utilized during request *z* handling, the reserved networking resources $y_{l,z}$ must be sufficient to ensure QoS requirement compliance related to network communication:
  $$y_{l,z}\geq Q_{K}\left(z\right)\cdot b_{l,z},\;\;\forall l\in L,\forall z\in Z$$
  where $b_{l,z}$ is a binary variable indicating whether link *l* is utilized during the request *z* handling process.
- For each network link *l* utilized during request *z* handling, the security level of the utilized network link must be at least at the required QoS defined for request *z*:
  $$s_{l}\geq Q_{s}\left(z\right)\cdot b_{l,z},\;\;\forall l\in L,\forall z\in Z$$
  where $b_{l,z}$ is a binary variable indicating whether link *l* is utilized during the request *z* handling process.

As the problem discussed in this paper is a joint task of QoS assurance and resource allocation, the selection criteria utilized in the resource allocation algorithm should depict the effects of resource allocation on the overall system’s load. One of the assumptions regarding the MEC infrastructure considered in this paper is that computational resources are all virtualized, and another is that network resources can be easily sliced and reserved for different types of requests. Following this assumption, the utilization of the standard deviation of the load as a fitness function for the resource allocation algorithm seemed reasonable, aimed at load balancing both computational and networking resources. Minimization of the average load promotes load distribution, which decreases the risk of bottlenecks and provides the resource allocation algorithm with a broader range of options for the deployment of new virtual machines (VMs) and containers in the network. This should result in better QoS assurance for a broader range of requests. While other metrics might have been utilized in the optimization process, e.g., energy efficiency or number of active servers, this might not have resulted in the desired resource allocation outcome. For instance, the minimization of active servers (resulting in lower energy consumption) will probably promote the execution of all requests on a single MEC server before activating any other servers. As a result, all containers or VMs will be deployed on a single server until we reach its total capacity before enabling another server for such deployment. However, such an approach could have disadvantages, because it will probably lead to an increased utilization of only selected network links leading to this server, while others will stay unused. Moreover, it could lead to a situation in which the closest and only server that can handle ultra-low latency service requests for some users becomes overloaded and cannot handle any requests, while other servers are too far away in the network infrastructure to assure proper handling of such requests. As such an outcome was not desired in the present research, the optimization criteria utilized in the resource allocation method discussed in this paper focused on load balancing the computational and networking resources available in the network, mitigating the risk of bottlenecks’ occurrence. To provide a better understanding of the proposed prediction-based resource allocation outcomes, additional metrics utilized for solution evaluation (and not as optimization criteria) might be utilized, e.g., the aforementioned energy consumption or request rejection rate. As this research focused on QoS assurance for handled requests, the request rejection rate was selected as the solution evaluation measure, as it directly showed how many requests could not be correctly handled. Additionally, in order to facilitate the resource algorithm analysis, the average load measure was also introduced and utilized in this research.

In order to assess the level of load balance in the network in line with the described approach, the load of each element of the network must be calculated first. Let $N_{i}$ indicate the i-th active node, $L_{j}$ indicate the j-th network link, and $Z_{k}$ indicate the k-th resource type, while *Z* is the set of resource types utilized in the system. For this setup, the following definitions are proposed:

- Computing resource load in an active node:

$$LOAD\left(N_{i},Z_{k}\right)=\frac{{\mathrm{USE}}_{\mathrm{COMP}}\left(N_{i},Z_{k}\right)}{{MAX}_{COMP}\left(N_{i},Z_{k}\right)}$$

where

$LOAD(N_{i},Z_{k})$—calculated load of the k-th resource in the i-th node;
${\mathrm{USE}}_{\mathrm{COMP}}\left(N_{i},Z_{k}\right)$—allocated resources of type $Z_{k}$ in node $N_{i}$;
${MAX}_{COMP}\left(N_{i},Z_{k}\right)$—total pool of resources of type $Z_{k}$ in node $N_{i}$.

- Network link load:

$$LOAD\left(L_{j}\right)=\frac{{\mathrm{USE}}_{\mathrm{NET}}\left(L_{j}\right)}{MAX_{\mathrm{NET}}\left(L_{j}\right)}$$

where

$LOAD(L_{j})$—calculated load of the j-th network link;
${\mathrm{USE}}_{\mathrm{NET}}\left(L_{j}\right)$—allocated bandwidth in network link $L_{j}$;
$MAX_{\mathrm{NET}}\left(L_{i}\right)$—total bandwidth of network link $L_{j}$.

- Set of all individual loads of links and active nodes:

$$\Omega =\left\{\begin{array}{c} LOAD\left(N_{1},Z_{1}\right),\;LOAD\left(N_{1},Z_{2}\right),\ldots ,\;\\ LOAD\left(N_{V},Z_{k}\right),\;\\ LOAD\left(L_{1}\right),\ldots ,LOAD\left(L_{E}\right) \end{array}\right\}$$

where

V—total number of active nodes;
E—total number of network links.

Given all introduced definitions, the following measures can be introduced:

- Average load measure. The value is calculated on the basis of individual loads of all network links and active nodes:
  $$\bar{LOAD}=\frac{1}{\left|\Omega \right|}\sum_{LOAD\epsilon \Omega }LOAD$$
- Standard deviation of the load, which is considered a load balance measure and a fitness function for the resource allocation algorithm. The smaller the value, the greater the load uniformity:
  $$\sigma =\sqrt{\frac{1}{\left|\Omega \right|-1}{\sum_{LOAD\epsilon \Omega }\left(LOAD-\bar{LOAD}\right)}^{2}}$$
- Request rejection rate measure, which will be utilized to evaluate the resource allocation method utilizing different prediction algorithms:
  $$Rejection\;Rate=\frac{Number\;of\;rejected\;requests}{Number\;of\;all\;requests}$$

In the proposed approach and the following experimentation, a single-objective optimization will be utilized in the resource allocation algorithm. The selected fitness function for this optimization algorithm is the defined standard deviation of the load, which as an optimization criterion should enforce load balancing between nodes and network links during the request handling process. The optimization criterion will be subjected to hard constraints for total resource allocation on each active node, each network link utilization limit, and each network link security capability according to the definitions provided in this section. Evaluation of the prediction-based resource allocation methods during the experimentation will be conducted utilizing two additionally defined measures: the average load measure and the request rejection rate measure. These two additionally defined measures for method evaluation were utilized as while standard deviation of the load is a good candidate for the fitness function utilized during per-request optimization, it does not provide an overview of the overall performance levels of the proposed methods; hence, additional measures must be utilized.

## 4. Proposed Methods

In order to ensure network stability and reliability, which are important factors that can affect system security, it is imperative to establish efficient service provisioning and effective handling of incoming network requests. Considering that the planned solution necessitates managing a queue of user requests over time, this process specifically entails routing individual end user requests while taking into account quality of service requirements for various traffic classes. Within the context of the network in question, characterized by a finite pool of available resources, it was posited that the resource allocation mechanism would synergize with the routing mechanism, aiming to achieve load balancing across the entire network system.

The following section introduces two prediction-based approaches, applying divergent techniques, where the first method uses Long Short-Term Memory (LSTM), a type of recurrent neural network (RNN), while the second relies on a statistical prediction technique utilizing an Autoregressive Integrated Moving Average (ARIMA) model. A metaheuristic in the form of a genetic algorithm was deployed as the baseline algorithm when utilizing the proposed methods, in accordance with the assumptions and metrics presented in the previous section, allowing for the comparative evaluation of the effectiveness of the proposed prediction approaches in different scenarios.

### 4.1. Baseline Algorithm

The baseline algorithm employs a genetic algorithm that iteratively explores a solution space represented by potential resource allocation configurations, resembling the principles of natural selection and evolution. Each iteration involves the generation of candidate solutions, which are submitted to selection, crossover, and mutation operators to produce offspring solutions. The efficiency of each solution is evaluated based on predefined load-balancing criteria.

The main concept of the proposed approach revolves around exploring a set of solutions represented by successive paths connecting vertices in the network graph, thereby implementing a routing mechanism and determining the routes for both uplinks’ and downlinks’ connections. A single solution is described by two genotypes, representing, respectively, the uplink and downlink connection paths using a list of successive nodes. Subsequently, within the algorithm, solutions are subjected to evaluation based on their routes. The algorithm operates in such a way that successive descendant populations achieve increasingly smaller values of this metric, striving to minimize the standard deviation of network loads. Additionally, the allocation mechanism extends to traffic management functionalities, which incorporate the ability to redirect traffic from leaf nodes where request initiation transpires to alternative leaf nodes. It also supports traffic segmentation along designated routes, enabling the division of the required communication resources into different paths. The proposed resource allocation mechanism can be seen in Algorithm 1.
**Algorithm 1:** Resource allocation mechanism
**Input:** Constraint graph, Allocation graph, Simulation parameters
**Output:** Allocation graph, Rejection rate, Average network load, Average standard deviation of network load1*Simulation state—*current stage of the simulation over time;2*Network traffic—*list of requests in queue form containing traffic history;3**while** *Simulation state < Simulation time* **do**4
**if** *Network traffic contains a handled request* **then**5

Release allocated resources for handled request;6
**end**7
**Receive** new request;8
**if** *allocation within a given leaf node is not possible* **then**9

Randomly select another available leaf node to switch request to;10

**if** *leaf switching is not possible due to insufficient resources* **then**11


**Add** rejected request to *Network Traffic*;/*/Reject pending request*12


**Increment** *Simulation state;*13


**Go to** 3;14

**end**15
**end**16
**Perform** *Initialization;*17
**Evaluate** *Population fitness;*18
*Epoch* ← *0;*19
**while** *Epoch < Number of epochs* **do**20

**Perform** *Selection, Crossover, Mutation;*21

**Evaluate** *Population fitness;*22

**Increment** *Epoch;*23
**end**24
*Evaluate best path* ← **Select** best routing path based on fitness population;25
*Splitted path score* ← **Evaluate** resource allocation fitness for the best paths;26
**if** *Splitted path score < Best path score* **and** *Splitted path is feasible solution* **then**27

**Update***Allocation graph*;28

**Add** request to *Network traffic;*29
**else**30

**if** *best routing path is feasible solution* **then**31


**Update***Allocation graph*;32


**Add** request to *Network traffic;*33

**else**34


**Add** rejected request to *Network traffic*;/*/Reject pending request*35

**end**36
**end**37
**Increment** *Simulation state;*38**end**39**Return** *Allocation graph, Rejection rate, Average network load, Average standard deviation of network load;*

### 4.2. Proposed Prediction-Based Methods

This approach shifts from reactive to proactive resource allocation by incorporating predictive analytics and is based on modeling the probability of traffic occurrence in successive time intervals. It involves analyzing the anticipated shares of individual traffic classes across these intervals. The percentage share of each traffic class not only provides an insight into the current network load but also enables the forecasting of future resource demand based on historical data. This stems from the assumption that each traffic class necessitates computational processes at the active node assigned to it. For example, if a certain traffic class is expected to represent 50% of the overall network traffic in a given time interval, implying its responsibility for half of the total traffic, then it is presumed that the node handling this traffic class will bear a load proportional to 50% of the requests from that class. Therefore, predicting the traffic share allows the prediction-based algorithm to enforce traffic routing restrictions on network elements whose resource demand is increasing. The proposed percentage share of a given traffic class can aid in understanding the potential load distribution on individual network elements, which may allow the network to be adequately prepared for incoming traffic, potentially mitigating the need to reject user requests. Hence, future load estimation is feasible based on the predicted shares of individual traffic classes, assuming that for each user request arriving into the network system, the class of that traffic is known and is associated with certain quality of service requirements. Preprocessing the collected synthetic network traffic data allowed us to divide incoming requests within time windows, consisting of 30 time units each. Statistics pertaining to the traffic distribution within each time interval were computed. The following Algorithm 2 preprocesses historical network traffic data to obtain traffic shares within specified time windows.
**Algorithm 2:** Preprocessing network traffic history data
**Input:** Traffic history, Time window, Start, End
**Output:** Traffic shares1*Traffic shares* ← empty list;2*Data* ← Extract data from *Traffic history* from *Start* to *End*;3*Segments* ← Iterate through *Data* and slice segments of size *Time window;*4**Foreach** *segment* **in** *segments* **do**5
**Calculate** unique classes and their count;6
**Calculate** class proportions;7
**Add** proportions to *Traffic shares;*8**end**9**Return** *Traffic shares;*

By utilizing these processed data, the future share of each traffic class was predicted. This prediction relied on the ten preceding statistics, from which the forecasted value of the eleventh statistic was derived. The algorithm responsible for predicting the next network traffic shares is presented in Algorithm 3. The dataset consists of network traffic requests categorized into different classes, namely video streaming, e-health, smart home, and autonomous vehicles. The gathered data are preprocessed to segment them into manageable time windows, facilitating subsequent analysis.
**Algorithm 3:** Network traffic share prediction
**Input:** Traffic history, Time window, Start, End, Lookback, 
**Output:** Traffic share prediction1*Traffic shares* ← Get *Traffic shares* using Preprocessing traffic history data algorithm;2*//Collect traffic shares from the last Lookback segments* *Previous traffic shares* ← *Traffic shares*[-Lookback:]; 3*Traffic share prediction* ← Generate output prediction for *Previous traffic shares* input;4**Return** *Traffic share prediction;*

#### 4.2.1. Prediction-Based Approach with ARIMA

Our method leverages Autoregressive Integrated Moving Average (ARIMA) modeling for prediction. ARIMA is a time-series analysis technique that captures temporal dependencies in data. In this context, the ARIMA model is trained on historical traffic data to forecast the future shares of different traffic classes, including the use of parameters such as autoregressive (AR), differencing (I), and moving average (MA), which are optimized to best fit the observed data. Once trained, the ARIMA model can predict future traffic shares based on past observations.

To properly prepare the ARIMA model, the data series must be stationary, which refers to the constancy over time of statistical properties within the dataset being analyzed. Based on the data, containing 2000 consecutive requests entering the network, the augmented Dickey–Fuller unit root test (ADF) was used to assess stationarity. At the confidence level of 5%, the hypotheses were formulated as follows:

- Null Hypothesis: The time series has a unit root, indicating that it is non-stationary;
- Alternate Hypothesis: The time series does not have a unit root, indicating that it is stationary.

The test results for the simulated data, comprising percentage statistics for the next 30 time units, are presented in Table 1. Based on the tests conducted for each dataset, which included traffic share statistics within a given time interval and a significance level of 5%, the hypothesis assuming non-stationarity of the data was rejected, and we accepted the alternative hypothesis of the stationarity of the time series.

Additionally, an analysis of autocorrelation and partial autocorrelation plots was conducted to select appropriate parameters *p*, *d*, and *q* for the ARIMA model. Based on the results of the statistical tests, which led to the rejection of the hypothesis of non-stationarity for each tested series, the parameter *d*, referring to the degree of differencing, should take a value of 0.

**Figure 6:**
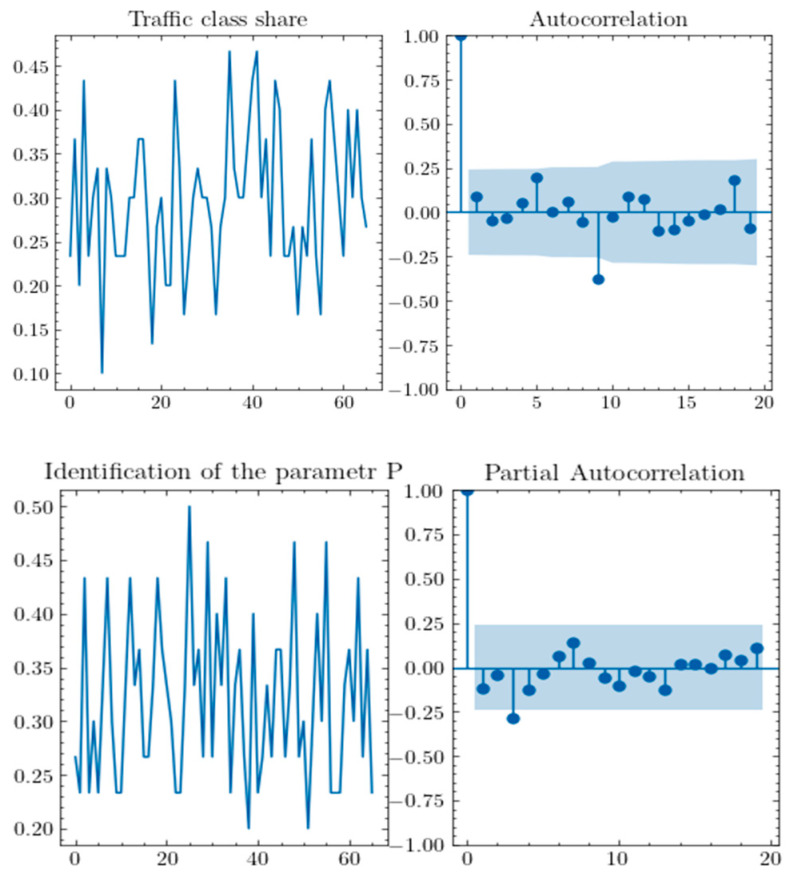
Time series ACF and PACF correlograms for autonomous vehicle traffic class.

The following is a formal analysis of time series for the traffic class associated with autonomous vehicles, exemplified by the examination of Autocorrelation Function (ACF) and Partial Autocorrelation Function (PACF) plots. The autocorrelation depicted in the plot below (Figure 6) suggests that the impact of delays beyond 9 is negligible, leading to the inference that an appropriate parameter *q* for this time series may be the value 9. Conversely, the analysis of the PACF plot indicates that the point at which partial autocorrelations lose significance is the value 8.

Due to the sinusoidal nature of the aforementioned autocorrelation plots, various combinations of parameters were iteratively tested to identify suitable parameters *(p, d, q)* based on the mean squared error (MSE) values. Selection of appropriate parameters for the ARIMA model, such as *p*, *d*, and *q*, was based on our analysis of historical data, and we used the grid search method to find the optimal values of these parameters. This process involves testing different combinations of parameter values and selecting those that best fit the data. The values selected through this process are shown in Table 2.

In Table 2, each row corresponds to a specific traffic class, and the values (*p*, *d*, *q*) indicate the parameters that were empirically determined to provide the best performance for the ARIMA model for that particular traffic class. The mean squared error (MSE) is also provided as a measure of the model’s accuracy in fitting the data. These empirically determined values serve as guidelines for configuring the ARIMA model to effectively predict future traffic patterns for each traffic class.

As part of the next step in the analysis of the ARIMA model, the following graph gives a visualization of the obtained prediction results for each traffic class, used in order to assess the accuracy of the model.

**Figure 7:**
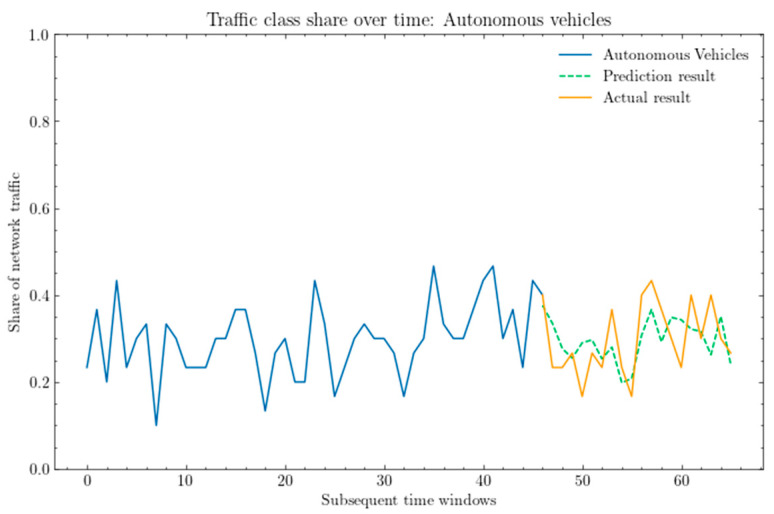
ARIMA model prediction results for traffic class: autonomous vehicles.

Figure 7 shows a comparison between the actual share of network traffic for the autonomous class and the predictions obtained using the ARIMA model.

In summarizing the above results, it can be inferred that the values do not perfectly reflect the share of traffic. The traffic share can range from 0 to 1, and it can be deduced that for autonomous vehicles, with an MSE value of 0.0051, the RMSE is approximately 0.0714, indicating that the difference between the predicted and actual values may vary by around 7.14%.

#### 4.2.2. Prediction-Based Approach with LSTM

This paper proposes using a Long Short-Term Memory (LSTM) neural network to capture the relationship between the preceding ten statistics that encompass the distribution of each network traffic class and thereby predict the subsequent—eleventh—statistic. The adoption of this model is motivated by its adeptness in capturing temporal dependencies within sequential data, a crucial aspect of forecasting time-series data.

In building a neural network for a given prediction objective, a bidirectional LSTM was chosen as the first layer to allow the model to incorporate both past and future information within the context of each input sequence. This enables a better understanding of temporal dependencies and more effective data analysis. The shape of the first layer (None, 10, 30), as shown in Table 3, implies that the first value denotes the batch size, which can be any number. Then, the second value signifies the sequence length, which represents ten time windows, containing ten statistics selected for prediction, and the third value indicates the feature size in each sequence. Bidirectional layers provide a robust framework for learning complex temporal patterns inherent in the data. The inclusion of a Dropout layer with a dropout rate of 0.01 serves to regularize the model and prevent overfitting by randomly deactivating a small fraction of neurons during training, thus promoting better generalization to unknown future data.

The Dense layer following the LSTM layer is responsible for aggregating information from the input sequences and transforming it into a response to a given prediction objective. In the proposed model, this layer reduces the output dimensionality from 20 to 5, corresponding to the number of traffic classes. In the final neural network model, the choice of linear activation function was driven by the assignment’s emphasis on predicting numerical values, aligning well with the nature of the problem.

Table 4 summarizes selected hyperparameters for the LSTM model. Additionally, when selecting the optimizer, both Adam and AdamW were considered, with AdamW proving more effective due to its ability to handle sparse gradients and counteract overfitting. By integrating Adam’s optimization benefits with weight decay regularization, AdamW offers enhanced generalization capabilities. Moreover, the decision to set a batch size was made to balance computational efficiency with training stability, facilitating frequent parameter updates without introducing a significant computational overhead. Finally, the adoption of the mean squared error loss function was motivated by its suitability for regression tasks, aiming to minimize the squared difference between predicted and actual values, thereby ensuring accurate predictions.

**Figure 8:**
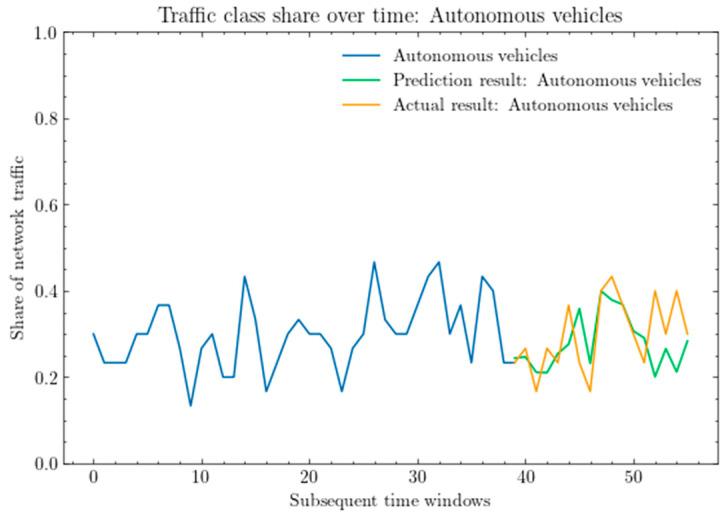
LSTM model prediction results for traffic class: autonomous vehicles.

Figure 8 shows a comparison between the actual share of network traffic for the autonomous class and the predictions obtained using the LSTM model. As shown, the prediction achieves high accuracy, but for the last intervals of the time windows, the forecast fails, predicting exactly the opposite trend.

In order to compare the effectiveness of the ARIMA and LSTM models in predicting different classes of network traffic, the mean squared error values for both models were analyzed across several scenarios. As shown in Table 5, the LSTM model achieved a better accuracy than ARIMA in most cases. Notably, LSTM outperformed ARIMA in scenarios such as video streaming, cloud gaming, and smart homes. However, ARIMA performed significantly better in the automotive scenario, while the e-health class showed almost no difference in performance between the two models.

While LSTM outperformed ARIMA in terms of MSE in most scenarios, LSTM requires larger datasets and a time-consuming training process. On the other hand, ARIMA requires data stationarity and model fitting, which are often difficult to achieve, especially for real-time data, which may not satisfy these assumptions or be sufficiently prepared for rapid prediction [33]. In our case, the use of the LSTM model also required more time; however, unlike ARIMA, it does not require retraining. This advantage, combined with its prediction accuracy for real-time applications, appears to justify the additional computational overhead.

The prediction results obtained from both the ARIMA and LSTM methods will be considered in the evaluation of the developed genetic algorithm. The proposed use of the prediction results involves calculating the difference between the latest actual statistics concerning the distribution of individual traffic classes within the network and the forecasted values. These differences, indicating increasing or decreasing shares of traffic classes over consecutive time intervals, will allow for precise adjustments to the fitness evaluation function values, particularly with high predictive accuracy.

## 5. Experimental Results

This section presents the specified configuration parameters and outcomes of the experiments conducted. The results underwent a qualitative analysis in which we interpreted their significance within the context of the research.

### 5.1. Configuration of Experiments

Our simulation followed the process shown in Figure 5. To conduct this study, a test dataset was created to represent network traffic data, along with the network infrastructure in the form of three network graphs differing in density.

The presentation of the conducted research requires the clarification of certain aspects. The fundamental issue is the traffic that is entering the network. This research utilized generated traffic data. The generated traffic, including resource requirements, was prepared to be as close to real-world examples as possible. However, the real-world use of edge computing is still limited; therefore, in many cases, assumptions had to be made on the basis of usage characteristics for each traffic type in currently used systems. For instance, in the case of automotive traffic, there are still very limited examples of car-to-car communication utilizing edge computing. During test data preparation, it was assumed that this type of traffic will mainly utilize sensor data exchange, similar to e-health data from medical devices, and that it will require more data processing. Similar assumptions on the basis of current use cases were made for the other traffic types. Table 6 outlines the percentage chance of a particular traffic class appearing in the inputs to the network in successive time intervals, as well as their required throughput and level of security within the QoS requirements. The level of security is represented by a scale from ‘D’ to ‘A’, where ‘A’ indicates the highest level of security. In the context of the network topology, this can be understood as preserving the properties of the wired connection on a given link.

Three different network bidirected graphs were utilized in the simulation research. The first of these was manually defined and configured according to Table 7 and comprises 11 nodes, including 3 computational nodes. The predefined graph represents a network in which all connections between nodes, i.e., links, have the highest security property, labeled ‘A’.

Computing resources, such as disk capacity, amount of RAM, and number of CUDA cores, were varied depending on the sets shown below in Table 8. Randomly selected nodes were designated as computational nodes, and computing resources from randomly chosen sets were assigned to these nodes.

The capacity of connections in the network was defined as a list of values, allowing for different capacities for different segments of the network. The final structure of the network was randomly selected, where the graph underwent a process of randomly selecting edges according to a specified density probability. Each selected link (edge) was assigned a capacity and security level based on previously defined parameters. Below, Table 9 shows relevant network parameters regarding the configuration of link properties.

In summary, proper configuration of the experiments required the consideration of various aspects, including network traffic characteristics, network topology properties, resources, and security requirements. Utilizing traffic queue simulation and determining QoS requirements enabled the precise definition of the research conditions.

### 5.2. Results of the Proposed Methods

This subsection presents the results of our simulations of the proposed prediction-based methods on different types of network topologies. As part of the evaluation, experiments were conducted for three different graphs and prediction methods within a simulation duration of 1000 time units. For each method, 20 simulations were performed within each graph. According to the metrics introduced in Section 3.3, load balancing, the network load, and the rejection rate were measured.

**Figure 9:**
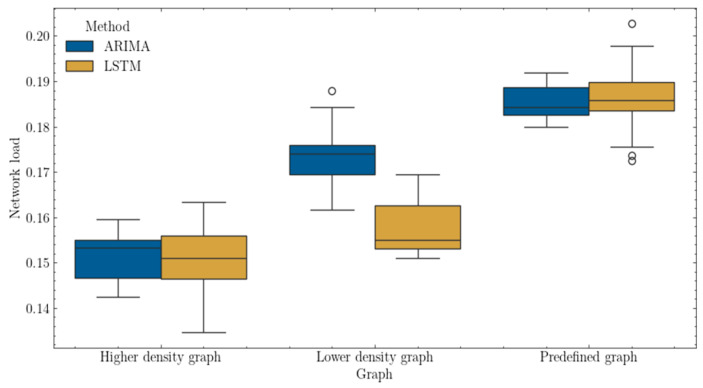
Network load statistics by method and graph.

Figure 9 presents a boxplot comparing the network load metric for different algorithms (ARIMA, LSTM) across different types of graphs (predefined, low-density, high-density). Intuitively, different graphs tend to achieve different average levels of network load due to their varying capabilities at handling heterogeneous traffic classes.

A predefined graph allows for traffic management with the highest level of connection security, defined as ‘A’. However, links available in other graphs may not be capable of handling requests from traffic classes with high QoS requirements when it comes to this security level aspect. Although the proposed methods, shown below, do not differ radically from each other, certain characteristics exhibit differences, such as a wider range of results for the recurrent neural network, especially for predefined and high-density graphs.

In analyzing the progression of individual iterations of the proposed approach, when obtaining a set of predicted shares for each of the five traffic classes in the next time window, depending on future growth, the fitness evaluation function rewards the exploration of connections between nodes with a decreasing future usage tendency and penalizes those with increasing demand, thus avoiding the formation of additional connections that may lead to excessive use of resources in the future.

**Figure 10:**
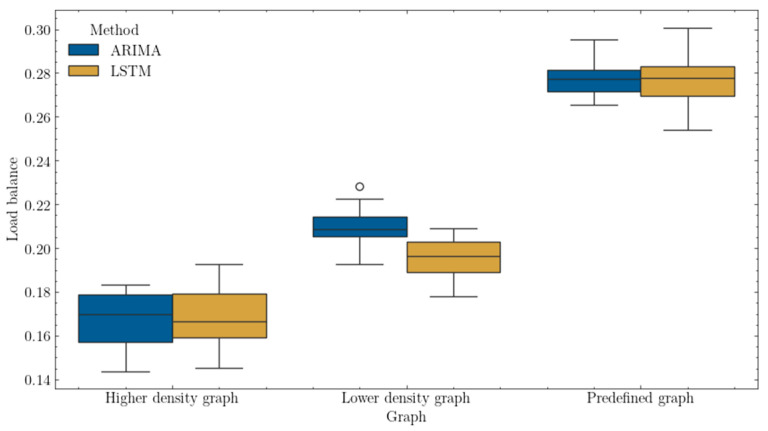
Load balance statistics by method and graph.

Figure 10 presents the load balance metric, which is the basis for evaluating the quality of a single solution, represented by an individual. Since the load balance is a measure that refers to the standard deviation of the load of the entire network, it can be assumed, based on the figure, that the best-balanced network, i.e., whose load is evenly distributed among the various elements, has a low-density graph that has been subjected to the LSTM method. For the other type of graphs, the distinction of differences is not so apparent.

**Figure 11:**
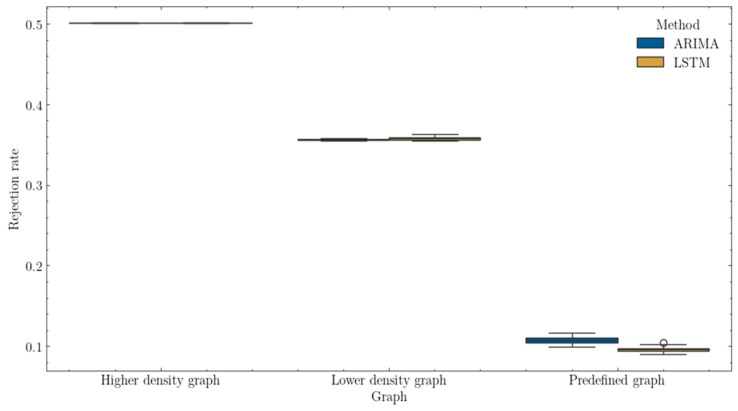
Rejection rate statistics by method and graph.

The last metric examined to compare the proposed methods is the rate of rejected requests. As shown in Figure 11, the rejection rates are almost identical for graphs representing different types of densities; meanwhile, for the predefined graph, it can be seen that the LSTM method achieved a relatively lower rejection rate.

A preliminary analysis of the reason for identical rejections revealed that networks with different levels of density rejected all requests within those traffic classes to which they could not provide an adequate level of security in its entirety. The rate of rejected requests shows how effectively the proposed methods perform under given conditions. However, it is important to consider that the characteristics of a specific network greatly influence request acceptance, particularly in cases where decisions regarding the considered traffic are binary—either the request may fully meet the quality-of-service requirements and be accepted, or it is not.

Further analysis of the normality of the distribution of the examined metrics, namely network load, load balance, and rejection rate, showed that the assumptions regarding a normal distribution were not met in at least one of these metrics within the examined classifying factors (independent variables): prediction method and graph type. Similarly, assumptions related to the homogeneity of variance were not met in all cases. Given the violation of a normal distribution and homogeneity of variance assumption, a non-parametric Kruskal–Wallis ANOVA test was utilized. This test is robust against violations of these assumptions and is appropriate for comparing multiple groups when the data do not meet the requirements for a two-way ANOVA test. It ranks the observations across all groups and examines whether there are significant differences in the distributions of the ranks between the groups, providing a reliable alternative to a parametric ANOVA. The results of the Kruskal–Wallis tests are presented below in Table 10 and provide comparative insights into the behaviors observed across the examined conditions. Hypotheses refer to the equality of mean ranks for consecutive populations and can be employed to ascertain whether there are statistically significant differences among two or more groups of independent variables.

In interpreting these results, it should be noted that for all three tests, the null hypothesis, stating that there are no differences between groups, was rejected. This indicates significant differences between group combinations for each metric.

Since the Kruskal–Wallis test does not indicate which specific groups exhibit differences, further analysis in the form of a post hoc test is necessary. The Conover test was used to determine which groups differed significantly from each other. Table 11 shows the results of the post hoc Conover test for the load balance metric.

Pairwise comparison shows that for all combinations of prediction methods and graph types, the null hypothesis may be rejected (*p* < 0.05), indicating that the compared groups are different, except for the comparisons between ARIMA and LSTM for the predefined graph type and higher-density graph. In this case, at the set level of significance, the null hypothesis was not rejected, suggesting no significant difference in the load balance metric between ARIMA and LSTM for a graph with a predefined highest possible security level of links, as well as for a graph with configured high-density links.

Further analysis of the differences present requires highlighting the nature of the synthetic data used in this study, which were generated according to the configuration of experiments shown in Table 5. Such generated data may lead to a certain unpredictability of the dataset and lack of seasonality. Although the obtained values of the evaluation metric for time-series prediction (MSE) were lower for the LSTM model, it is necessary to analyze the implications of the use of these prediction models for the differences in the results achieved.

**Figure 12:**
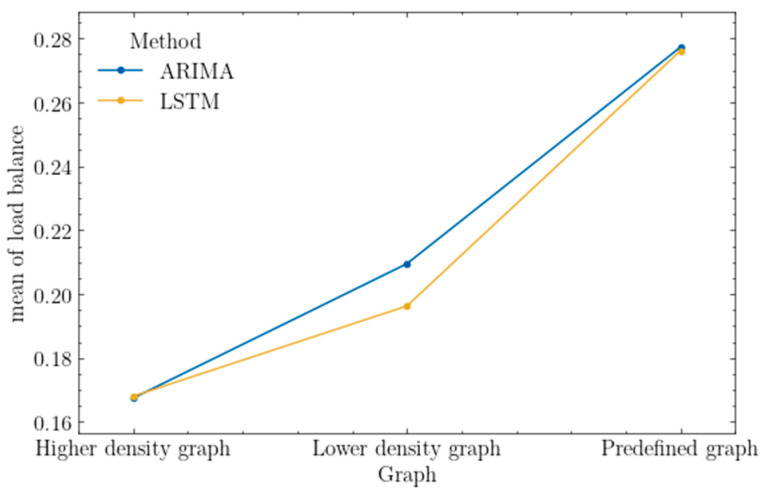
Interaction plot for average of load balance under two classifying factors.

As shown in the interaction graph below (Figure 12), the ARIMA model achieved a slightly higher value of the load balance metric for the low-density graph by approximately 3%, a difference deemed significant according to the test results. For the other types of graphs, there were no significant differences observed in the results.

Table 12 shows the results of the post hoc Conover test for the network load metric, which yielded identical results for both rejecting the null hypothesis and failing to reject the null hypothesis, similar to the previous tests. Regardless of the method used, the results for the high-density graph do not differ, similar to the predefined graph. Differences can be observed for the low-density graph, and also for each method across different types of graphs. Once again, the high-density graph highlights the issues of both algorithms utilizing network resources for users’ requests. The best results are achieved for the predefined graph, which can likely be attributed to its inherent highest possible security level properties spanning the entire network.

**Figure 13:**
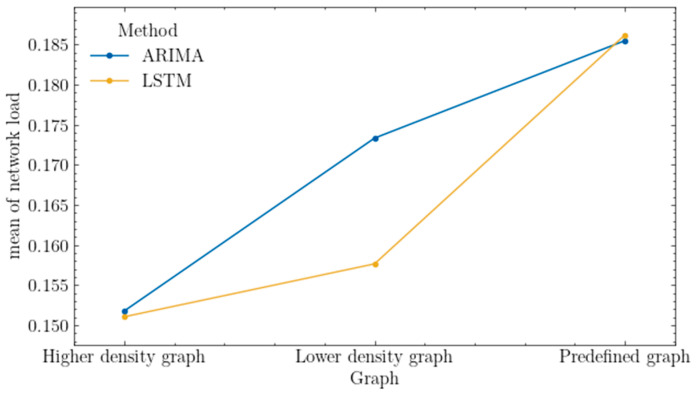
Interaction plot for average network load under two classifying factors.

The reliability of the results is also depicted by the interaction plot for factor-level statistics in Figure 13. According to the tests, the ARIMA model achieved a higher value of the network load metric by about 15%, which is assumed to be significant.

The network load provides information about the current state of the network. For identical traffic, in the experiments conducted, the lower average load compared to the LSTM model may suggest that resource allocation based on this model leads to less traffic being processed. It is worth noting the potential impact of the algorithm on path length, as this directly correlates with increased resource usage.

As can be seen in Figure 12 and Figure 13, for both the mean network load and mean load balance, the utilization of the ARIMA network traffic prediction model for the low-density network can be characterized by an increased risk when resources are over-allocated (considered as allocating more resources that are actually needed due to incorrect prediction) on network elements that are critical to handling requests from a particular class of traffic, such as those with high security requirements. The relatively small number of links in such a network can lead to the existence of few combinations of feasible routes for requests. If the prediction is incorrect, the resource allocation algorithm may direct traffic to these critical network elements, leading to the over-allocation of resources and ignoring unused nodes and links (which could have been viable for handling actual traffic), resulting in an increased network load and a reduced load balance. Finally, this can result in the rejection of further requests over time, caused by overloading critical network elements.

The last evaluated metric for the Conover test is the rate of rejected requests, and the results of this test are shown in Table 13. Based on the results, it can be concluded that there are statistically significant differences in the rejection of requests between different prediction models and graph types. In the case of the ARIMA model, significant differences exist between different graph types (higher density, lower density, predefined), as for the LSTM model. An exceptional case occurs when considering a high-density graph for combinations of two methods, ARIMA and LSTM, where no significant differences were found between these pair of groups.

**Figure 14:**
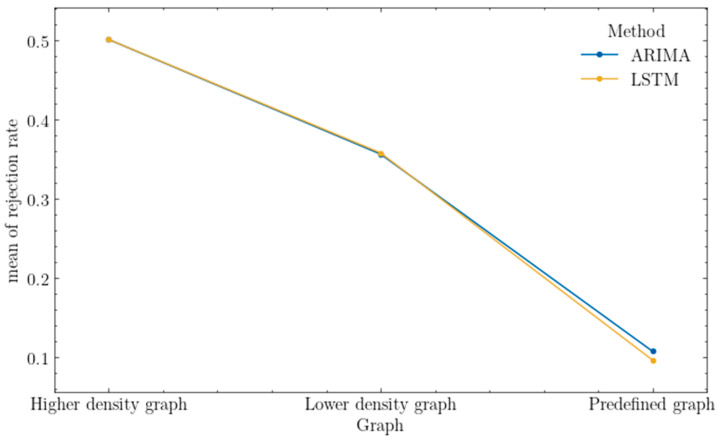
Interaction plot for average rejection rate under two classifying factors.

Although in Figure 14, the graphical differences between methods for the low-density graph are not noticeable, the post hoc test revealed that the differences in this case are statistically significant. For the predefined graph, the method utilizing the LSTM model resulted in a lower rejection rate by 10% compared to using the ARIMA model. The prediction mechanism affects the avoidance of nodes and links that are in the vicinity of a node with increasing resource demands. In a network where the highest security level is guaranteed and there is no risk of failing to meet security requirements, differences in the rejection rate are more apparent. The impact on rejection rates may be due to the accuracy of LSTM predictions, which were higher than ARIMA. It is worth noting that in some situations, correct prediction may not necessarily lead to significant performance gains and improved resource allocation. For example, in the case of high-density networks, there were conditions in which requests from a certain traffic class could never be fulfilled, due to the inability to provide a guaranteed level of security along the entire length of the network’s routes. Ultimately, these requests were always rejected. When there is only one type of possible rejection, since the highest level of security is always guaranteed, as in the case of a predefined graph, the LSTM method is more effective than the ARIMA model, managing to more accurately predict subsequent traffic statistics.

**Figure 15:**
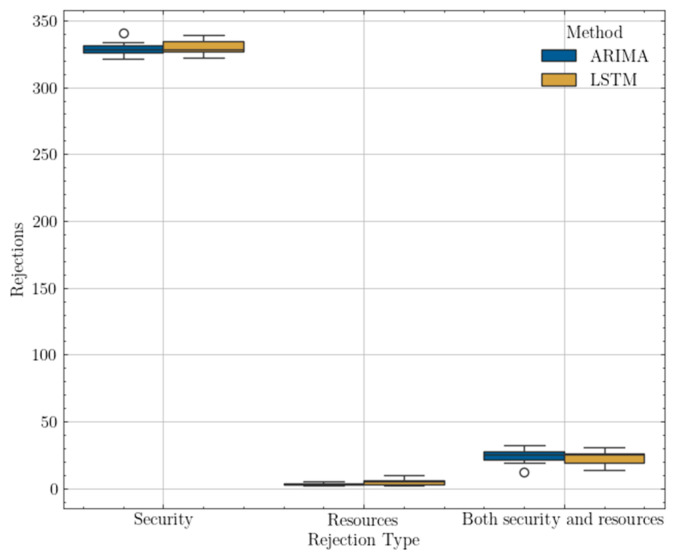
Statistics of rejected requests and rejection types for a low-density graph depending on the method.

In order to highlight additional differences occurring for the low-density graph, Figure 15 presents box plots based on the rejection reason (*rejection type*) and proposed methods, where the *Y*-axis represents the number of rejections.

Despite the LSTM method achieving a higher effectiveness for the predefined graph, measured by the rejection rate of requests, it was found that for a low-density graph, the ARIMA method was slightly more effective. In this particular case, the overall average number of rejections was lower by just under 1% compared to the LSTM model. The number of rejections due to a failure to meet security requirements for simulations using the ARIMA model was only 0.2% lower than its alternative. However, a substantial difference can be observed in rejected requests resulting from inadequate communication and computational resource fulfillment, where LSTM exhibits a 30% higher rejection rate. Conversely, ARIMA demonstrates higher rejection rates for requests failing to meet both categories of requirements. The above result may confirm previous considerations regarding the risk of inefficient resource allocation in the case of incorrect predictions of the ARIMA model, as the prediction results were inferior to those of the LSTM model. It is true that LSTM rejects more requests due to security requirements; however, the number of rejected requests for both security and resource reasons is much lower.

The proposed prediction objective is to forecast the demand for specific computing resources at nodes and the throughput of links, which may not ensure adequate security for users’ requests entering the network. There is a need for additional research, which would complement the above results with additional information. As part of that research, it is worthwhile engaging in forecasting, taking into account the security parameter, to investigate how the manner in which request requirements are fulfilled can be controlled, and the impact of the proposed solutions on the main efficiency metric of resource allocation, which is in this case the final number of rejected requests. Understanding how these two approaches affect request handling effectiveness is crucial for better evaluating and comparing their efficiency and practical application. Additional research can provide valuable insights that will allow for a better understanding of the complex factors influencing the decision-making process regarding the selection of the appropriate predictive model under various network conditions.

## 6. Conclusions

The main goal of this paper was to discuss the challenges, prospects, and potential solutions that can be implemented in next-generation network-based systems in order to ensure an increased reliability in network communication and data processing while optimizing resource allocation.

This paper has proposed a prediction-based resource allocation approach developed to ensure the fulfillment of the required QoS for various types of IoT use cases and autonomous adaptability to changes in end users’ requests, based on request analysis and machine learning methods optimizing the utilization of available resources. The proposed approach defines a graph-based next-generation network model, which can be effectively utilized for resource allocation in networks with MEC capabilities. We have set out a resource allocation process utilizing this model and both network traffic prediction and heuristic resource allocation to ensure the reliability and security of systems deployed in such infrastructures. The experimental verification has shown a discernible impact observed in environments characterized by top-tier network security standards across all connections, as represented within a predefined network graph. In this case, despite LSTM and ARIMA models showing a statistically similar average network load and balance, LSTM exhibited a significantly better performance in rejection rates, allowing us to reduce the necessity of rejecting users’ requests by 10%. Under ideal network security conditions, LSTM is a more effective method than ARIMA, as evidenced by the simulations. 

While the discussed results provide valuable information on the behavior of the proposed method and algorithms, they mainly enable further research that can delve into specific aspects of both traffic prediction and resource allocation in accordance with the proposed network model. Such research may lead to novel solutions in this field. The experimentation also shows that security concerns are a key factor in the process of resource allocation, which leads to the conclusion that these aspects of NGN systems should be analyzed jointly in order to develop novel algorithms and methods.

Further research conducted based on the findings discussed in this paper should focus on extending the set of QoS parameters considered in the prediction-based resource allocation problem and developing new heuristic-based research allocation algorithms that can offer a broader utilization of additional data provided by the traffic prediction methods. Also, interesting research could be conducted on refining the model’s performance under varying network conditions and exploring potential extensions or adaptations to the model. Finally, as mentioned in this paper, the proposed approach can be verified in a more complex network scenario, which could utilize network segment encapsulation.

## Figures and Tables

**Table 1:** Results of the ADF statistical test regarding the stationarity of the series for simulated data concerning five traffic classes.

Traffic Class	*p*-Value	Hypothesis Verification
video streaming	$2.67\times 10^{-8}$	Reject *H*_0_ and accept *H*_1_
cloud gaming	$5.03\times 10^{-12}$	Reject *H*_0_ and accept *H*_1_
e-health	$4.24\times 10^{-14}$	Reject *H*_0_ and accept *H*_1_
smart home	$8.66\times 10^{-11}$	Reject *H*_0_ and accept *H*_1_
autonomous vehicles	$7.53\times 10^{-3}$	Reject *H*_0_ and accept *H*_1_

**Table 2:** Empirically selected values (*p*, *d*, *q*) for the ARIMA model.

Traffic Class	Values (*p*, *d*, *q*)	MSE
video streaming	(7, 0, 8)	0.00573
cloud gaming	(5, 0, 2)	0.00300
e-health	(1, 0, 5)	0.00088
smart home	(9, 0, 5)	0.00402
automotive	(6, 0, 9)	0.00485

**Table 3:** Long Short-Term Memory sequential neural network model.

Layer (Type)	Output Shape	Parameter
Bidirectional (LSTM)	(None, 10, 30)	2520
Dropout	(None, 10, 30)	0
Bidirectional (LSTM)	(None, 20)	3280
Dense	(None, 5)	105
Activation	(None, 5)	0

**Table 4:** LSTM hyperparameters.

Hyperparameter	Value
Number of hidden layers	3
Dropout rate	0.01
Batch size	5
Activation function	Linear
Optimization solver	AdamW
Learning rate	0.001
Number of epochs	250
Test data split	30%
Loss function	Mean squared error

**Table 5:** Comparison of prediction error (MSE) for ARIMA and LSTM models.

Traffic Class	MSE (ARIMA)	MSE (LSTM)
video streaming	0.0082	0.0069
cloud gaming	0.0048	0.0032
e-health	0.0010	0.0012
smart home	0.0070	0.0062
automotive	0.0051	0.0075

**Table 6:** Probabilities of occurrence of a given traffic class with a predefined throughput and security level requirements.

Traffic Class	Occurrence Probability	Security QoS	Throughput QoS
E-health	5%	‘A’	30 Mbps
Video streaming	30%	‘D’	150 Mbps
Cloud gaming	20%	‘D’	50 Mbps
Smart home	15%	‘C’	20 Mbps
Automotive	30%	‘B’	30 Mbps

**Table 7:** Initial configuration for the examined network graphs.

Graph Type	No. of Nodes	Link Density Ratio	No. of Links	Computational Node Ratio	No. of Computational Nodes
Predefined graph	11	N/A	36	N/A	3
Lower-density graph	20	0.1	72	0.3	5
Higher-density graph	20	0.2	92	0.3	4

**Table 8:** Sets of resources for computing nodes.

Set	Storage	RAM	VRAM	Cores	CUDA Cores
No. 1	4000	256	256	256	800
No. 2	1000	64	64	32	400

**Table 9:** Edge properties with their occurrence probabilities.

Type	Value	Occurrence Probability for Lower-Density Graph	Occurrence Probability for Higher-Density Graph
Security level	‘A’	75%	30%
	‘B’	25%	20%
	‘C’	0%	30%
	‘D’	0%	20%
Capacity	1000 Mbps	25%	25%
	3500 Mbps	25%	25%
	5000 Mbps	25%	25%
	10,000 Mbps	25%	25%

**Table 10:** Kruskal–Wallis tests results indicating the existence of differences between the combinations of groups of independent variables (Prediction Method, Graph Type) and the given dependent variables (Metric).

Prediction Method	Graph Type	Metric	H-Statistic	*p*-Value
LSTM	Predefined graph	Network load	106.32	<0.001
LSTM	Lower-density graph			
LSTM	Higher-density graph	Load balance	97.00	<0.001
ARIMA	Predefined graph			
ARIMA	Lower-density graph	Rejection rate	113.84	<0.001
ARIMA	Higher-density graph			

Indicates significant difference at α = 0.05.

**Table 11:** Post hoc Conover pairwise comparison of load balance metric across prediction models and given graph types.

Method × Graph	ARIMA × Higher-Density Graph	ARIMA × Lower-Density Graph	ARIMA × Predefined Graph	LSTM × Higher-Density Graph	LSTM × Lower-Density Graph	LSTM × Predefined Graph
ARIMA × Higher-density graph	1	<0.001	<0.001	1	<0.001	<0.001
ARIMA × Lower-density graph	<0.001	1	<0.001	<0.001	<0.001	<0.001
ARIMA × Predefined graph	<0.001	<0.001	1	<0.001	<0.001	1
LSTM × Higher-density graph	1	<0.001	<0.001	1	<0.001	<0.001
LSTM × Lower-density graph	<0.001	<0.001	<0.001	<0.001	1	<0.001
LSTM × Predefined graph	<0.001	<0.001	1	<0.001	<0.001	1

Indicates significant difference at α = 0.05.

**Table 12:** Post hoc Conover pairwise comparison of network load metric across prediction models and given graph types.

Method × Graph	ARIMA × Higher-Density Graph	ARIMA × Lower-Density Graph	ARIMA × Predefined Graph	LSTM × Higher-Density Graph	LSTM × Lower-Density Graph	LSTM × Predefined Graph
ARIMA × Higher-density graph	1	<0.001	<0.001	1	0.009	<0.001
ARIMA × Lower-density graph	<0.001	1	<0.001	<0.001	<0.001	<0.001
ARIMA × Predefined graph	<0.001	<0.001	1	<0.001	<0.001	1
LSTM × Higher-density graph	1	<0.001	<0.001	1	0.006	<0.001
LSTM × Lower-density graph	0.009	<0.001	<0.001	0.006	1	<0.001
LSTM × Predefined graph	<0.001	<0.001	1	<0.001	<0.001	1

Indicates significant difference at α = 0.05.

**Table 13:** Post hoc Conover multiple comparison of the rejection rate metric across prediction models and given graph types.

Method × Graph	ARIMA × Higher-Density Graph	ARIMA × Lower-Density Graph	ARIMA × Predefined Graph	LSTM × Higher-Density Graph	LSTM × Lower-Density Graph	LSTM × Predefined Graph
ARIMA × Higher-density graph	1	<0.001	<0.001	1	<0.001	<0.001
ARIMA × Lower-density graph	<0.001	1	<0.001	<0.001	<0.001	<0.001
ARIMA × Predefined graph	<0.001	<0.001	1	<0.001	<0.001	<0.001
LSTM × Higher-density graph	1	<0.001	<0.001	1	<0.001	<0.001
LSTM × Lower-density graph	<0.001	<0.001	<0.001	<0.001	1	<0.001
LSTM × Predefined graph	<0.001	<0.001	<0.001	<0.001	<0.001	1

Indicates significant difference at α = 0.05.

## Data Availability

The data used in this research are available upon request to the authors.
